# Characteristics and genesis of permian chert in the middle and upper Yangtze Region, China

**DOI:** 10.1371/journal.pone.0323122

**Published:** 2025-10-17

**Authors:** Da-zhi Zhang, Jin-xiong Luo, Bin Feng, You-bin He, Zhan Wen

**Affiliations:** 1 Exploration and Development Research Institute of Daqing Oilfield Company Ltd, Daqing, China; 2 School of Geosciences, Yangtze University, Wuhan, China; 3 Changqing Division, China National Logging CO. LTD, Xi’an, Shaanxi, China; 4 Hulunbuir Branch, Daqing Oilfield Company Ltd, Daqing, China; China University of Mining and Technology, CHINA

## Abstract

Cherts, as chemical sedimentary formations, serve as repositories of historical evolutionary data encompassing paleotectonics, paleogeography, and paleoclimate. Furthermore, they play a crucial role as geological foundations for oil and gas exploration. In the Upper Yangtze region, the origin and underlying genesis mechanisms of unstratified cherts from the Permian period have been subject to ongoing debate. This study employs lithological analyses including outcrop profiles and thin-section observations alongside geochemical analyses of macronutrients, trace elements, and rare earth elements to investigate the depositional environment of laminated cherts from the Permian era. Additionally explored are the siliciclastic origins of non-laminated cherts and the diagenetic mechanisms at play in this area. The findings indicate that stratified chert in the Middle and Upper Yangtze regions originate from basin sedimentation below the carbonate compensation depth interface while unstratified chert primarily form through dissolution of carbonates attributable to both hydrothermal activity and seawater processes. This comprehensive investigation provides a robust geological foundation for oil and gas exploration within this study area while also serving as a valuable reference for future research on studies related to chert.

## Introduction

Cherts are chemical sedimentary rocks that can be categorized into two groups based on their formation and morphological differences: laminated chert and unstratified chert [1,2]. Laminated cherts primarily develop independently or interbedded with mudstones [3–5], while unstratified cherts occur as nodules, agglomerates, or bands within carbonates or plutons [6]. Throughout geological history, both siliciclastic sediments and cherts have been widely distributed but were particularly abundant during specific epochs such as the Eocene siliciclastic succession [7,8] and the Permian siliciclastic depositional events of the United Paleozoic [6,9]. Due to their high density and stable physical/chemical properties along with resistance to weathering processes, different genetic types of cherts provide significant records of paleotectonic/paleogeographic/paleoclimatic/ paleoceanographic evolutionary information during geohistorical periods [10–13]. Moreover, radiolarian-rich chert associated with mudstone/shale formations often exhibit a high potential for organic matter/hydrocarbon accumulation leading to the formation of large-scale oil/gas fields [14,15]. Exploration samples from locations like the Domanik field in Russia’s Burazo Basin [16] and Lower Ordovician Penglaiba Formation in Tarim Basin [17] confirm this phenomenon. Therefore, studying these materials provides crucial geological insights for hydrocarbon exploration purposes especially in terms of hydrocarbon source rock prediction.

The Middle and Upper Yangtze region is located in the south of China, which generally refers to Sichuan, Chongqing, Guizhou, Yunnan, Hubei, Hunan and other regions, covering an area of about 50 × 10^4^ km^2^. It is not only the main production area of conventional natural gas in China, but also the key area of Marine shale gas exploration and development in China in the past two decades. Abundant unstratified cherts of Permian are extensively distributed in the Upper Yangtze region, China [2]. However, in recent years, the study of Paleozoic in the Middle and Upper Yangtze area mainly focuses on the exploration potential of Carboniferous shales, sedimentary facies characteristics of Lower Cambrian, sedimentary facies characteristics of Sinian Dengying Formation [18,19], Ordovician shale exploration potential [20], Cambrian shale exploration potential [21], and so on. But the genetic mechanisms and origin of siliciclastic deposits remain highly contentious [2,22–24]. The hydrothermal genesis is widely acknowledged for laminated cherts, some of which exhibit a biogenic nature [6,24–30].

In order to address these aforementioned constraints comprehensively, this study integrates petrological and geochemical characteristics using sedimentological and geochemical methods previously reported in literature to analyze the sedimentary environment and origin of cherts in the Lower Yangtze region. The aim is to elucidate the genesis mechanism of each type of chert while providing insights into their depositional environments and establishing a geological foundation for Permian oil and gas exploration in the Yangtze region.

## Geological background

**Fig 1:**
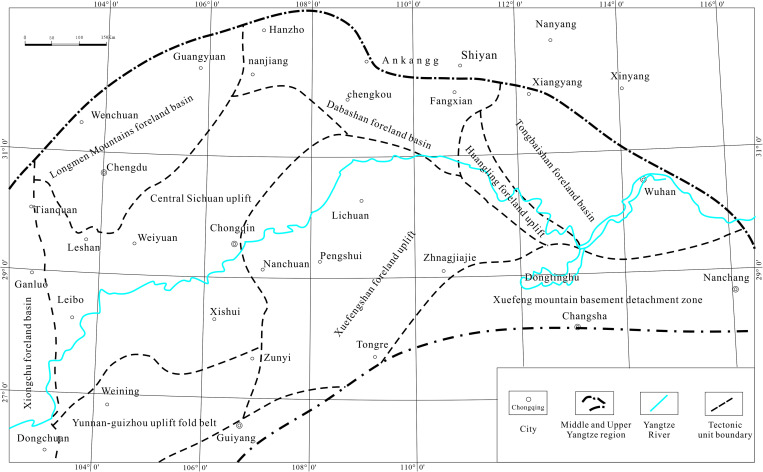
Location of the Middle and Upper Yangtze region.

The Middle and Upper Yangtze regions in southwestern China (Fig 1) are bordered by Baoxing-Xingjing-Zhaojue-Liupanshui to the southwest, Baoxing-Guangyuan-Hanzhong to the northwest, Hanzhong-Xiangyang-Jiujiang to the north, and Xiushui-Yueyang-Changde Jishou-Guiyang to the southeast. This region encompasses eight provinces and cities: Yunnan, Guizhou, Sichuan, Chongqing, Shaanxi, Xiang, and Gan. It spans longitudes 102°–116°E and latitudes 26°–33°N over an area of approximately 50 × 10^4^ km^2^.

The Permian in the Middle-Upper Yangtze region is widely distributed, exhibiting thicknesses ranging from 400 to 800 m. It can be categorized into Upper Permian systems as well as Middle and Lower Permian systems. Specifically, within our study area, we observe the presence of the Permian Qixia and Maokou Formations, along with the Upper Permian Wujiaping and Changxing Formations. However, it should be noted that a limited occurrence of the Lower Permian system is observed solely in the Majiaoba area of Jiangyou in northwest Sichuan.

## Data and methods

A total of 43 outcrop sections within the study area were meticulously surveyed, described, and sampled for lithological analysis. Based on this comprehensive analysis, a total of 31 samples were collected to determine their chemical composition, macronutrient content, trace element presence, and concentrations of rare earth elements (REEs). Geochemically speaking, these samples exhibited exceptional freshness without any signs of alteration or calcite infills; moreover, they displayed minimal weathering effects. The samples underwent meticulous in-house crushing to obtain a fine powder with particles sized at 200 mesh without any risk of contamination before being carefully dried in an oven. Subsequently, the sample analysis was conducted by the Institute of Geology and Geophysics, Chinese Academy of Sciences, which allows individuals or organizations to carry out public welfare collaborative research.

### Chert types and characteristics

Various types of cherts were formed during the Permian period in the Middle-Upper Yangtze region and can be categorized based on their formation into nodular, agglomerate, banded, and thin-bedded cherts.

### Nodular, agglomerate, and banded cherts

**Fig 2:**
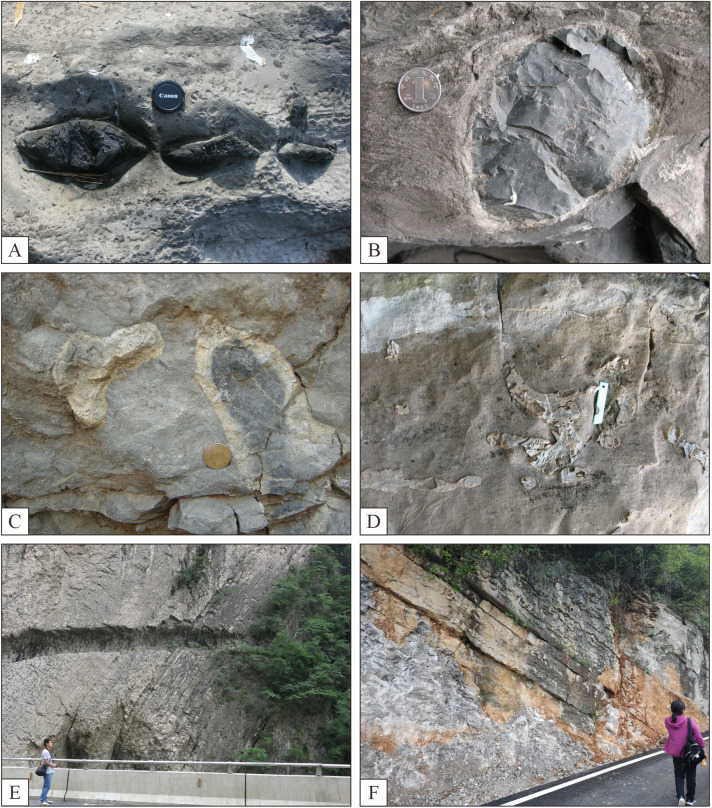
Features of unstratified silica. A-nodular siliciclastic rock, siliceous nodules cis-layered, Wujiaping Formation, Gaolu, Xuanen, Hubei. B-Nodular silica, Maokou Formation, Songkan, Tongzi, Guizhou; C-Glomerate silica, Wujiaping Formation, Daxiakou, Xingshan, Hubei; D-Glomerate silica, Changxing Formation, Songkan, Tongzi, Guizhou; E-Striped silica, Wujiaping Formation, Miaoba, Chengkou, Chongqing; F-Striped silica, Wujiaping Formation, Lianziya, Zigui, Hubei.

Nodular (Fig 2A), agglomerated (Fig 2B), agglomerated (Fig 2C) and irregular ginger-like forms (Fig 2D), commonly referred to as flint nodules, agglomerates, and bands, predominantly exhibit shades of gray-black or gray-white (Fig 2B), resembling concentric mosaics (Fig 2C). These structures frequently manifest in nodular (Fig 2A), ellipsoidal (Fig 2B), agglomerated (Fig 2C), lenticular, irregular ginger-like forms (Fig 2D), as well as banded patterns (Figs 2E,F).

**Fig 3:**
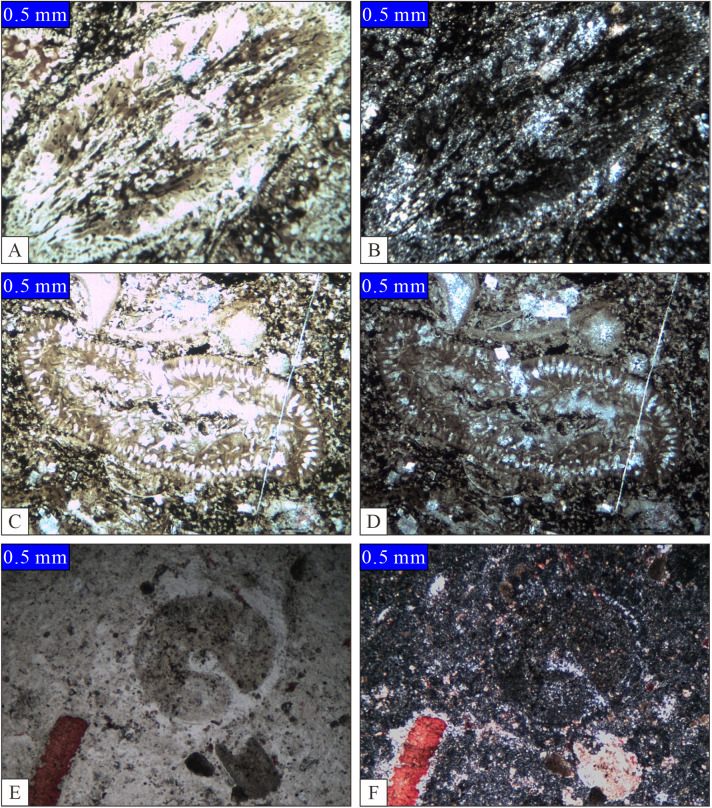
Microscopic characteristics of unstratified siliciclastic rocks. A-bioclastic silica, silicified nepheline algae, Changxing Formation, Songkan, Tongzi, Guizhou, single polarized light; B-same as A, orthogonal polarized light; C – biogenic clay-bearing silica, green algae, Mao Kou Formation, Songkan, Tongzi, Guizhou, single polarization; D – same as C, orthogonal polarization; E-bioclastic silica, bioclastic, Wujiaping Formation, Daxiaokou, Xingshan, Hubei, single polarized; F-same as E, orthogonal polarized.

The thickness of the nodules typically ranges from a few centimeters to up to twelve centimeters, with aspect ratios generally below 5. They are distributed parallel to the facies and vertically isolated in relation to the facies direction (Figs 3A, B, C). The striped silica exhibits thin to medium laminations with an aspect ratio ranging from 5–20 and displays notable stratification characteristics (Figs 3E, F). It maintains a roughly uniform thickness but may exhibit varying degrees of undulation at different levels; occasionally it presents honeycomb-like features due to calcareous dissolution within its structure (Figs 3E, F).

The mineral composition of nodules, agglomerates, and banded silicas primarily comprises chalcedony and microcrystalline quartz while frequently incorporating mud, organic matter, residual calcite, and dolomite as subsequent additions. The presence of grayish-white silica may be attributed to dolomitization (Fig 3).

Additionally, nodulars, agglomerates, and banded chert frequently preserve complete fossils as well as fossil fragments such as algae (Fig 3A,B,C,Dera), brachiopods, echinoderms, and sponges (Fig 3E,F), which is in line with the preservation of fossils in limestone peridotites.

### Thin-layered chert

**Fig 4:**
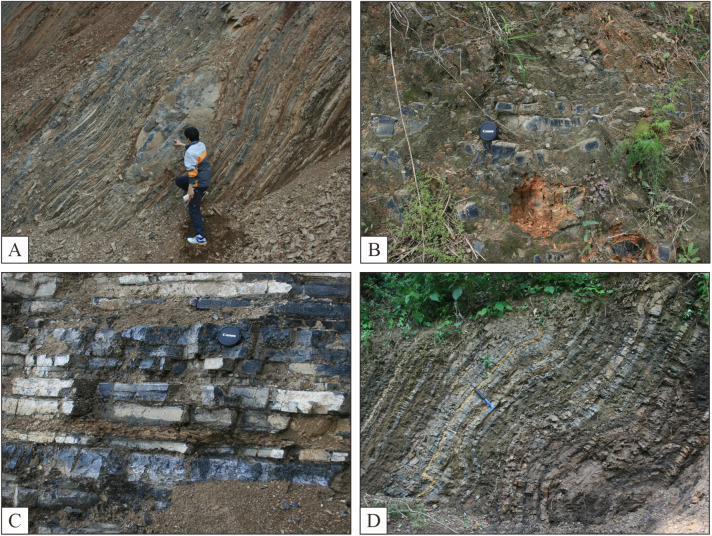
Characteristics of thin-layered silica. A-Wujiaping Formation, Qiaosan, Jingshan, Hubei; B-Changxing Formation, Shazi, Yanhe, Guizhou; C-Dalong Formation, Changjianggou, Guangyuan, Sichuan; D-Dalong Formation, Shuanghui, Wangcang, Sichuan.

The thin-bedded silicas are predominantly distributed within the Middle Permian Maokou Formation and Upper Permian Wujiaping, Changxing, and Dalong Formations (Fig 4). They primarily occur as thin beds, although a few exhibits medium bedding with horizontal stratification, generally contemporaneous with siliceous mudstone, siliceous shale, mudstone, and shale. Additionally, they partially coincide with thinly to moderately bedded or lenticular limestone (Fig 4A).

**Fig 5:**
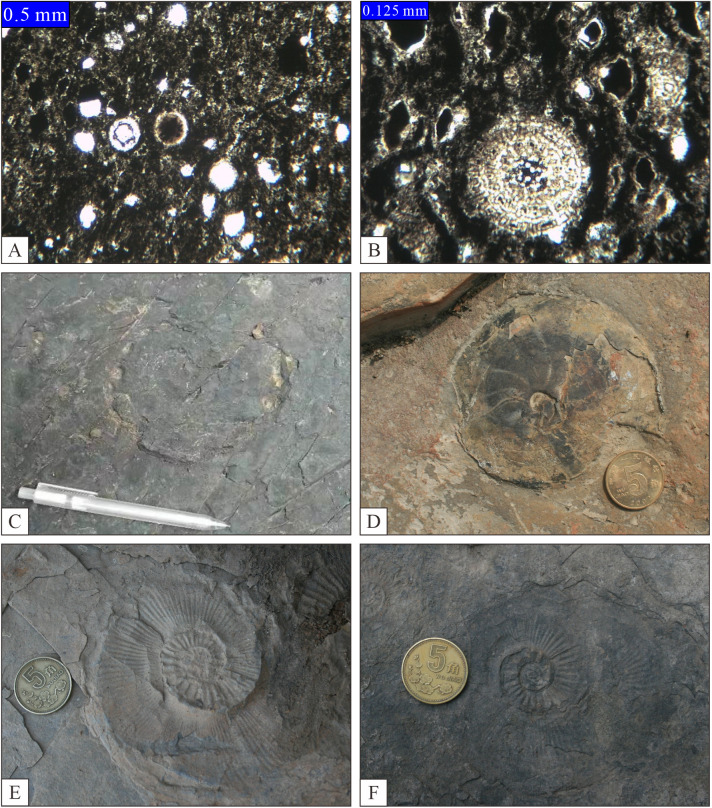
Paleontological features of stratified silica. A-Radiolite, Dalong Formation, Wangcang Shuanghui, Sichuan; B-Radiolite, Dalong Formation, Wangcang Shuanghui, Sichuan; C-Ammonite, Wujiaping Formation, Liujiachang, Songzi, Hubei; D-Ammonite, Dalong Formation, Qiaosan, Jingshan, Hubei; E-Ammonite, Dalong Formation, Guankou, Qingchuan, Sichuan; F-Ammonite, Wangcang Shuanghui, Sichuan.

The thin-bedded silica is typically characterized by its dark, gray-black to black coloration, high density, and exceptional hardness. Additionally, it frequently exhibits rhyolitic lamination in conjunction with mudstone and shale (Figs 4A, B, C, and D). Within the thin-layered silica deposits (Fig 5), two primary categories of biological fossils were identified: microfossils predominantly comprising radiolarians (Figs 5A, B), and macrofossils encompassing chrysolites (Figs 5C, D, E, F), small-bodied brachiopods with delicate shells as well as mesozones, bivalves, and foraminifera associated with planktonic or swimming life forms. This phenomenon was observed to be relatively prevalent within the study area.

### Distribution characteristics of thin-layered chert

Based on lithological analysis, the outcrop sections in the study area were examined to ascertain the planar distribution of thin cherts from the Permian Maokou, Wujiaping, and Changxing Formations. Additionally, contour maps depicting the content of these thin cherts were compiled.

**Fig 6:**
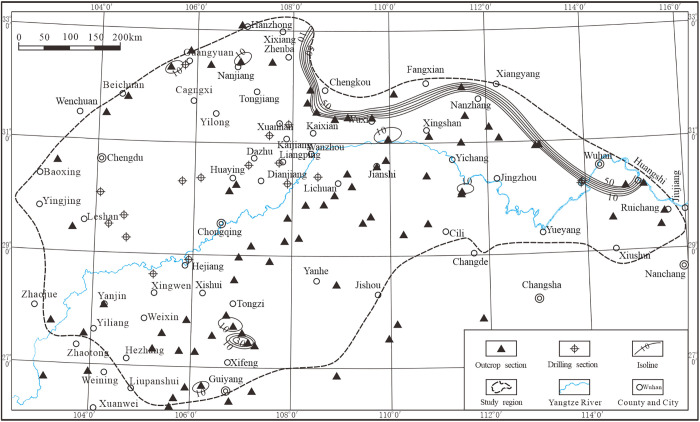
Thickness contours of the Maokou Formation stratified silica in the Middle and Upper Yangtze region.

The chert of the Mao Kou Formation, characterized by its thin-layered structure (Fig 6), is predominantly distributed in the northeastern region of the study area, spanning from Xixiang to Huangshi. It exhibits a distinct banding pattern and displays variable element content ranging from 10% to 50%. Additionally, sporadic occurrences of this rock type can be found near Guangyuan, Nanjiang, Tongzi, and Guiyang, with elemental concentrations typically ranging from approximately 10% to as high as 30%.

**Fig 7:**
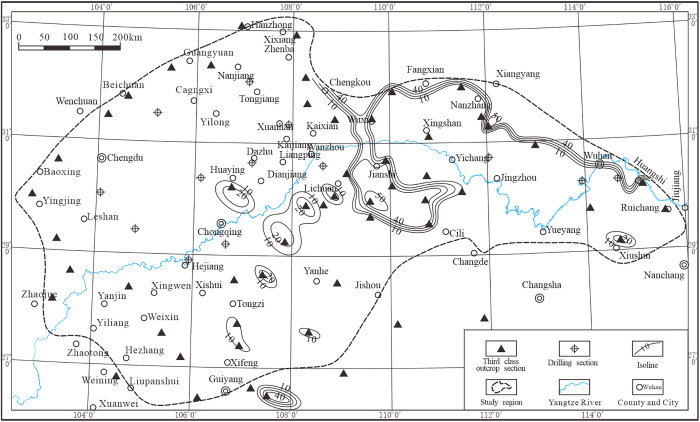
Thickness contours of the Wujiaping Formation stratified silica in the Middle and Upper Yangtze region.

The chert of the Mao Kou Formation, characterized by its thin-layered structure (Fig 7), is predominantly distributed in the northeastern region of the study area, spanning from Xixiang to Huangshi. It exhibits a distinct banding pattern and displays variable element content ranging from 10% to 50%. Additionally, sporadic occurrences of this rock type can be found near Guangyuan, Nanjiang, Tongzi, and Guiyang, with elemental concentrations typically ranging from approximately 10% to as high as 30%.

**Fig 8:**
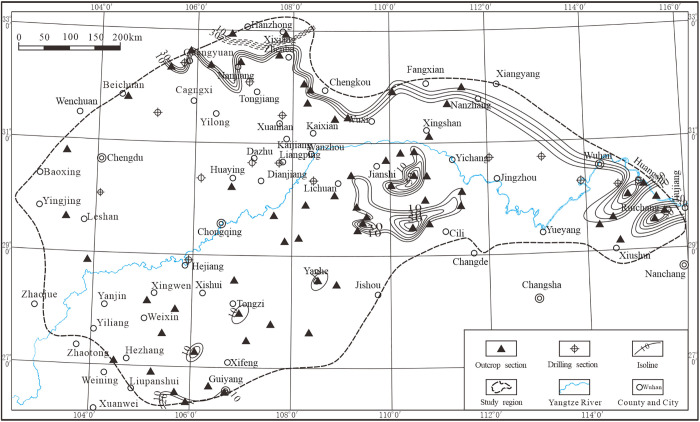
Thickness contours of the Changxing Formation stratified silica in the Middle and Upper Yangtze region.

The thinly bedded cherts of the Changxing Formation (Fig 8) exhibit significant scaling and are widely distributed across the study area, ranging from Guangyuan in the north to Jiujiang in the east, with element content varying between 10% and 30%, reaching up to 50%. Additionally, scattered distributions were observed in the Jiansi-Wufeng, Enshi-Shimen, Yanhe, Tongzi, and Wejin areas, with element content ranging from 10% to 50%.

### Geochemical characteristics

Geochemical analysis is crucial for investigating the origin of siliciclastic rocks, typically relying on major element, trace element, and REE content as well as their ratios. In this study, 31 samples were selected based on their chemical compositions (Tables 1 and 2), trace element data (Table 3), Al/(Al + Fe + Mn) statistics (Table 4), and REE analysis (Table 5).

**Table 1:** Major element contents of Permian chert in measured sections in the Middle-Upper Yangtze region.

Section name	Sample type	Sample name	Major earth element content %										Total content %	Fe/Ti	(Fe+Mn)/Ti	Al/(Al+Fe+Mn)
			SiO_2_	TiO_2_	Al_2_O_3_	FeO+Fe_2_O_3_	MnO	MgO	CaO	Na_2_O	K_2_O	P_2_O_5_				
Changjianggou, Guangyuan, Sichuan	Nodular	P2m–17–3	93. 14	0. 02	0. 11	0. 73	0. 08	0. 26	2. 60	0. 07	0. 06	0. 01	100. 47	42. 33	47. 72	0. 09
		P2m–24–3	91. 91	0. 01	0. 03	0. 72	0. 08	0. 49	3. 51	0. 02	0. 02	0. 01	100. 49	64. 61	72. 89	0. 03
		P2m–32–3	84. 83	0. 02	0. 11	0. 95	0. 12	1. 02	6. 38	0. 02	0. 06	0. 06	100. 37	54. 83	62. 36	0. 07
		P2m–35–3	86. 12	0. 02	0. 05	1. 01	0. 10	1. 04	5. 25	0. 02	0. 05	0. 08	100. 43	62. 40	69. 60	0. 03
		P2m–37–2	81. 84	0. 02	0. 08	0. 90	0. 08	0. 60	8. 19	0. 01	0. 05	0. 08	100. 37	54. 77	60. 14	0. 06
		P3w–3–3	89. 43	0. 03	0. 19	0. 76	0. 07	0. 33	4. 37	0. 04	0. 05	0. 01	100. 25	28. 75	31. 82	0. 14
		P3w–8–3	92. 68	0. 03	0. 07	0. 76	0. 08	0. 67	2. 42	0. 06	0. 06	0. 01	100. 42	33. 13	36. 93	0. 06
	Thin layer	P3d–5–3	95. 55	0. 01	0. 02	0. 72	0. 09	0. 38	1. 27	0. 05	0. 04	0. 01	100. 12	85. 94	98. 20	0. 02
Lishihe, Xuanhan, Sichuan	Nodular	P2m–14–3	91. 06	0. 02	0. 02	0. 82	0. 09	0. 48	3. 74	0. 08	0. 04	0. 02	100. 36	60. 01	67. 20	0. 02
		P2m–16–3	85. 99	0. 02	0. 04	0. 85	0. 09	0. 21	6. 49	0. 04	0. 05	0. 03	100. 50	62. 57	69. 99	0. 03
		P3w–5–2	97. 64	0. 01	0. 02	0. 71	0. 09	0. 08	0. 74	0. 02	0. 04	0. 02	100. 22	66. 13	75. 30	0. 02
		P3w–6–5	96. 44	0. 01	0. 02	0. 62	0. 07	0. 36	1. 18	0. 03	0. 04	0. 01	100. 22	68. 06	76. 47	0. 02
		P3w–8–2	84. 11	0. 03	0. 22	1. 02	0. 13	1. 35	6. 47	0. 05	0. 12	0. 26	100. 37	35. 29	40. 38	0. 12
		P3w–11–2	73. 68	0. 02	0. 02	0. 77	0. 07	0. 42	13.46	0. 01	0. 03	0. 04	100. 46	56. 68	62. 66	0. 02
		P3w–16–3	84. 65	0. 01	0. 02	0. 78	0. 08	1. 86	6. 01	0. 02	0. 03	0. 03	100. 42	73. 76	82. 33	0. 02
		P3w–17–3	99. 21	0. 01	0. 01	0. 41	0. 06	0. 06	0. 11	0. 03	0. 02	0. 02	100. 34	75. 23	88. 47	0. 02
		P3w–21–3	86. 64	0. 02	0. 03	0. 68	0. 09	0. 11	6. 76	0. 03	0. 04	0. 01	100. 42	45. 68	52. 15	0. 03
		P3w–28–2	87. 04	0. 02	0. 01	0. 63	0. 07	0. 12	6. 56	0. 03	0. 04	0. 01	100. 22	46. 38	52. 33	0. 01
		P3w–30–3	69. 61	0. 01	0. 01	0. 73	0. 07	0. 36	16.07	0. 00	0. 03	0. 04	100. 34	66. 48	73. 44	0. 01
Jianshan Wuxi, Chongqing	Nodular	P2m–20–1	91. 11	0. 01	0. 16	0. 50	0. 05	0. 09	4. 57	0. 02	0. 05	0. 02	99. 88	82. 21	91. 31	0. 18
		P3w–11–1	72. 18	0. 01	0. 21	0. 29	0. 04	0. 16	14.98	0. 02	0. 07	0. 08	99. 61	38. 00	43. 30	0. 32
		P3w–15–1	52. 79	0. 01	0. 11	0. 35	0. 02	0. 23	25.87	0. 02	0. 03	0. 09	99. 73	50. 72	54. 43	0. 18
		P3w–19–1	92. 00	0. 02	0. 36	1. 47	0. 15	0. 11	3. 20	0. 04	0. 11	0. 08	99. 84	113. 81	126. 98	0. 14
		P3w–29–1	57. 41	0. 00	0. 07	0. 18	0. 02	0. 19	23.49	0. 02	0. 03	0. 02	99. 61	52. 50	59. 60	0. 20
Shuanghui, Wangcang, Sichuan	Nodular	P2m–19–2	98. 04	0. 00	0. 07	0. 96	0. 10	0. 08	0. 30	0. 04	0. 02	0. 01	99. 87	373. 85	417. 75	0. 04
		P2m–20–1	76. 83	0. 00	0. 03	0. 44	0. 04	0. 43	12.22	0. 03	0. 02	0. 02	99. 35	168. 95	186. 17	0. 05
Average value			85. 18	0. 01	0. 08	0. 72	0. 08	0. 44	7. 17	0. 03	0. 05	0. 04	—	75. 50	84. 61	0. 0
	FeO+Fe_2_O_3_ indicates the total iron content, calculated as [(Fe_2_O_3_%×112/160)+(FeO%×56/72)]×160/112.															

**Table 2:** Chemical composition of Permian chert in measured sections in the Middle-Upper Yangtze region.

Lithology		SiO_2_	TiO_2_	Al_2_O_3_	Fe_2_O_3_	MnO	MgO	CaO	Na_2_O	K_2_O	P_2_O_5_	FeO	Number of samples
Nodular, agglomerate banded silica	Maximum value	99.21	0.03	0.36	0.97	0.15	1.86	25.87	0.08	0.12	0.26	1.02	30
	Minimum value	52.79	0	0.01	0	0.02	0.06	0.11	0	0.02	0.01	0.12	
	Average value	84.65	0.01	0.08	0.51	0.08	0.44	7.39	0.03	0.05	0.04	0.55	
Thin-layered silica		95.55	0.01	0.02	1.01	0.09	0.38	1.27	0.05	0.04	0.01	0.87	1

**Table 3:** Trace element contents of Permian cherts in measured sections in the Middle-Upper Yangtze region.

Section Name	Lithology	V	Ni	Cr	Co	Cu	Zn	V(V + Ni)	V/Cr	Ni/Co	Cu/Zn
P3d-2–2	Micrite	29.33	28.5	28.5	4.8	8.9	189.08	0.52	1.03	5.63	0.05
P3d-6–3	Micrite	1384	79.1	79.1	9.12	66.9	157.36	0.89	17.5	19.2	0.43
P3d-7–2	Micrite	15.15	14.6	14.6	3.12	16.3	31.06	0.51	1.04	4.58	0.52
P3d-6–4	Thin-layered silica	274.66	54.5	54.5	6.61	64.11	64.07	0.83	4.11	8.25	0.99

**Table 4:** Al/(Al + Fe + Mn) statistics of Permian chert in measured sections in the Middle-Upper Yangtze region.

Lithology	Al/(Al+Fe+Mn)			Number of samples
	Maximum value	Minimum value	Average value	
Nodular, agglomerate, banded silica	0.43	0.01	0.09	30
Thin-layered silica		0.14	0.14	1

**Table 5:** Rare earth element contents of Permian chert in measured sections in the Middle-Upper Yangtze region.

Section name	Sample type	Sample name	Rare earth element content (%)														ΣREE	LREE/HREE	Ce/Ce*	Eu/Eu*
			La	Ce	Pr	Nd	Sm	Eu	Gd	Tb	Dy	Ho	Er	Tm	Yb	Lu				
Changjianggou, Guangyuan, Sichuan	Nodular	P2m-17-3	0.26	0.45	0.05	0.19	0.04	0.01	0.03	0.01	0.03	0.01	0.02	0	0.02	0	1.02	0.92	0.84	1.58
		P2m-24-3	0.16	0.3	0.04	0.15	0.03	0.01	0.03	0	0.03	0.01	0.02	0	0.02	0	0.78	0.69	0.85	1.13
		P2m-32-3	1.4	1.24	0.22	0.83	0.15	0.03	0.16	0.03	0.17	0.04	0.09	0.01	0.08	0.01	4.46	0.74	0.48	0.95
		P2m-35-3	2.58	1.58	0.36	1.5	0.35	0.05	0.28	0.04	0.26	0.06	0.15	0.02	0.13	0.02	7.39	0.8	0.34	0.72
		P2m-37-2	2.47	1.78	0.39	1.48	0.36	0.05	0.29	0.04	0.27	0.06	0.16	0.02	0.13	0.02	7.52	0.8	0.39	0.73
	Thin layer	P3w-3-3	0.53	0.88	0.12	0.46	0.12	0.02	0.08	0.01	0.09	0.02	0.06	0.01	0.05	0.01	2.44	0.72	0.77	0.97
		P3w-8-3	0.32	0.59	0.07	0.28	0.07	0.02	0.07	0.01	0.08	0.02	0.05	0.01	0.05	0.01	1.64	0.5	0.85	0.13
		P3d-2-4	2.37	3.57	0.49	2.01	0.49	0.09	0.41	0.07	0.43	0.09	0.22	0.03	0.18	0.02	10.46	0.74	0.72	0.84
		P3d-5-3	0.16	0.23	0.04	0.13	0.03	0.02	0.03	0	0.03	0.01	0.02	0	0.02	0	0.72	0.74	0.68	3.04
		P3d-6-4	4.24	5.58	0.76	3..10	0.82	0.19	1.01	0.19	1.33	0.31	0.89	0.13	0.85	0.13	19.53	0.35	0.67	0.9
Lishihe, Xuanhan, Sichuan	Nodular	P2m-14-3	1.47	1.49	0.27	1.04	0.27	0.03	0.17	0.03	0.15	0.03	0.08	0.01	0.06	0.01	5.1	1.06	0.51	0.69
		P2m-16-3	1.1	0.97	0.21	0.88	0.24	0.04	0.23	0.04	0.22	0.05	0.13	0.02	0.12	0.02	4.27	0.55	0.43	0.76
		P3w-5-2	0.75	1.27	0.18	0.7	0.14	0.02	0.13	0.02	0.11	0.02	0.06	0.01	0.05	0.01	3.46	0.84	0.74	0.72
		P3w-6-5	0.46	0.56	0.11	0.37	0.06	0.01	0.07	0.01	0.06	0.01	0.04	0.01	0.04	0.01	1.8	0.74	0.54	0.89
		P3w-8-2	3.27	4..02	0.66	2.73	0.59	0.13	0.67	0.11	0.65	0.13	0.36	0.05	0.3	0.04	13.7	0.6	0.59	0.89
		P3w-11-2	0.77	1.23	0.19	0.72	0.15	0.03	0.15	0.02	0.15	0.03	0.09	0.01	0.08	0.01	6.63	0.6	0.7	0.73
		P3w-16-3	1.12	1.03	0.22	0.89	0.2	0.04	0.24	0.04	0.22	0.05	0.13	0.02	0.12	0.02	4.31	0.53	0.45	0.8
		P3w-17-3	0.88	0.38	0.21	0.79	0.16	0.03	0.19	0.02	0.13	0.02	0.06	0.01	0.05	0.01	2.94	0.79	0.19	0.76
		P3w-21-3	0.92	1.1	0.18	0.74	0.16	0.04	0.2	0.03	0.19	0.04	0.1	0.02	0.09	0.01	3.82	0.56	0.58	0.86
		P3w-28-2	1.66	1.83	0.38	1.52	0.38	0.06	0.34	0.06	0.33	0.06	0.018	0.03	0.16	0.02	7	0.64	0.51	0.77
		P3w-30-3	1.84	1.88	0.43	1.9	0.51	0.11	0.65	0.1	0.56	0.1	0.26	0.03	0.2	0.03	8.6	0.52	0.46	0.8
Jianshan Wuxi, Chongqing	Nodular	P2m-20-1	0.56	0.63	0.16	0.62	0.14	0.03	0.15	0.03	0.14	0.03	0.09	2	0.08	0.02	2.7	0.44	0.46	0.75
		P3w-11-1	2.45	1.61	0.58	2.29	0.44	0.08	0.48	0.08	0.39	0.08	0.21	0.04	0.22	0.044	8.98	0.63	0.29	0.77
		P3w-15-1	2.29	2.29	0.56	2.32	0.49	0.1	0.53	0.09	0.49	0.1	0.3	0.05	0.27	0.04	9.93	0.54	0.44	0.86
		P3w-19-1	2.72	2.3	0.7	2.93	0.66	0.12	0.65	0.11	0.56	0.12	0.29	0.05	0.27	0.04	11.53	0.61	0.36	.0.80
	Nodular	P3w-29-1	3.82	2.7	0.89	3.64	0.73	0.14	0.77	0.12	0.62	0.12	0.32	0.05	0.28	0.04	14.25	0.7	0.32	0.82
Shuanghui, Wangcang, Sichuan	Nodular	P2m-3-1	1.19	1.96	0.29	1.1	0.21	0.04	0.23	0.04	0.17	.0.04	0.1	0.02	0.09	0.02	5.5	0.67	0.73	0.72
		P2m-19-2	0.1	0.1	0.02	0.08	0.02	0	0.02	0	0.01	0	0	0.01	0	0	1.37	0.46	0.47	0.66
		P2m-20-1	0.17	0.1	0.05	0.19	0.04	0.01	0.04	0.01	0.02	0.01	0.02	0.01	0	0	0.67	0.64	0.23	1.48
Songkan, Tongzi, Guizhou	Nodular	P2q-11-1	0.1	0.1	0.05	0.17	0.03	0	0.03	0.01	0.01	0.01	0.01	0.01	0	0.01	0.54	0.16	0.27	0.63
		P2m-28-2	0.4	0.15	0.11	0.38	0.06	0.01	0.07	0.01	0.03	0.01	0.01	0.01	0.01	0.01	1.29	0.67	0.16	0.66
Shigudong, Xuanhan, Sichuan	Nodular	FYY-1	2.32	3.66	0.53	2.63	0.69	0.18	0.9	0.14	0.8	0.16	0.38	0.05	0.28	0.04	12.76	0.53	0.72	0.99
		YG-3	1.87	0.73	0.19	1.66	0.45	0.11	0.44	0.07	0.41	0.08	0.2	0.03	0.15	0.02	6.41	0.59	0.24	1.08
		YGD-3	2.2	3.03	0.49	2.69	0.83	0.25	1.32	0.18	0.91	0.16	0.33	0.04	0.19	0.02	12.64	0.57	0.63	1.01
		YGD4-3	5.97	8.73	1.18	5.47	1.17	0.24	1.32	0.18	0.91	0.16	0.33	0.04	0.19	0.02	25.91	0.97	0.71	0.84
Average value			1.57	1.71	0.32	1.38	0.32	0.07	0.35	0.05	0.31	0.06	0.16	0.02	0.14	0.02	6.52	0.65	0.53	0.94

Analysis of the chemical composition of silica (Tables 1 and 2) reveals that SiO_2_ dominates the chemical compositions of nodular, agglomerate, banded, and thin-layered silica. The SiO_2_ content in nodular, agglomerate, and banded silica ranges from 44% to 99.55%, with an average value of 83.14%, while CaO content ranges from 0.11% to 26.57%, with an average value of 7.98%. Thin-layered silica has a SiO_2_ content of 83.77% and a CaO content of only 5.97%. Compared to nodular, agglomerate, and strip silica, thin-layered silica has lower CaO content but higher P_2_O_5_ content. Trace element analysis (Table 3) shows that the contents of a few trace elements in silica rocks differ considerably from the crustal mean, ranging from several to tens of times the crustal mean. The overall Ba, As, Zn, Zr, and Sr contents were high, while the Co, Ni, Rb, Cs, Th, and U contents were low; other trace element contents did not differ significantly. Trace element Co/Ni and Th/U values were low: 0.08 and 0.21 on average.

An REE analysis showed (Table 5) that the total amount of REEs in the silica ranges from 0.37 to 25.91 (10−6), with an average of 6.36, and is widespread. The REE content and total amount of silica in the Maokou Formation are lower than those in the Wujiaping Formation. The REE contents of the silica rocks show that the δCe values range from 0.19 to 0.85, with an average of 0.53 and a significant negative anomaly; the δEu values range from 0.66 to 3.04, with a large variation and average of 0.97; and the light REE (LREE)/ heavy REE (HREE) values range from 0.44 to 1.06, with an average of 0.67.

The REE analysis revealed (Table 5) that the silica samples exhibit a wide range of total REE concentrations, varying from 0.37 to 25.91 (10−6), with an average value of 6.36. It is noteworthy that the distribution of REEs in these samples is extensive. Comparatively, both the REE content and total silica amount in the Maokou Formation are lower than those observed in the Wujiaping Formation. The δCe values for the silica rocks display a significant negative anomaly, ranging from 0.19 to 0.85 (average: 0.53). Similarly, there is considerable variation in δEu values (ranging from 0.66 to 3.04; average: 0.97). Additionally, the LREE/HREE ratios span between 0.44 and 1.06 (average: 0.67).

## Discussion

### Genesis of thin-layered chert

The depositional environment of the thin cherts in the study area is inferred to be deep-water based on our analysis. Radiolarian cherts have been discovered at Guangyuan Changjiang Gou, Guangyuan Gediba, Guangyuan Guankou, and Wangcang Shuanghui in northwest Sichuan during the Dalong Formation, providing compelling evidence for investigating their depositional environment. Radiolarian cherts are commonly associated with deep-sea deposits, as supported by numerous studies on modern and ancient radiolarians [6,31–33]. Therefore, it is likely that the study area represents a basin environment. The primary justifications for this assertion are as follows.

Firstly, thin cherts commonly contain radiolarian fossils. Based on the analysis of radiolarian cherts, it can be inferred that the sedimentary environment of these rocks in the study area is likely to be deeper, below the calcite compensated depth (CCD) interface. However, it should be noted that the CCD interface exhibits spatial and temporal variations. In modern times, the CCD interface generally occurs at depths ranging from 4000 to 5000 meters and reaches its maximum depth in the hypertoxic zone of the equatorial Pacific [34]. At relatively high latitudes and closer to continents, this interface tends to become shallower due to regional factors. Prior to the Late Jurassic period, calcareous organisms were not as abundant in paleo-oceans compared to their presence in modern oceans; moreover, siliciclastic organisms faced less competition during that time (Ellingsen et al., 2024). Consequently, it can be concluded that the CCD interface was significantly shallower in paleo-oceans than what is observed today.

Following the Late Jurassic period, the CCD interface experienced a decline to 2500 m [35], or even below 4000 m due to the sudden proliferation of calcareous planktonic foraminifera and super violet plankton. Consequently, the thin-layered chert in the study area often coexists with thin- to middle-layered mud-crystal limestone or is interspersed with lenses of mud-crystal limestone. This observation suggests that the deep-water Formation of the thin-layered chert in this study area is closely associated with and situated beneath the CCD interface.

Secondly, apart from the commonly found radiolarian fossils, the thin-layered chert in the study area also contains biological fossils such as chrysolites and foraminifera. Yang (1992) conducted a study on chrysolites from the Upper Permian of southern China and concluded that they inhabited a deeper water basin at an approximate depth of 200 m [36]. Wang (1993), in their investigation of foraminifera in Permian radiolarian cherts from the Suwan region [37], determined that these organisms were deposited in a deep-water terrarium environment. These findings suggest that both foraminifera and foraminifera associated with radiolarians predominantly originate from deep-water depositional environments.

Thirdly, the thin chert monoliths in the study area are typically a few centimeters to several dozen centimeters thick and frequently interbedded with siliceous shales, mudstones, and tuffs, resulting in the formation of rhyolites (Fig 4). The well-developed fine horizontal laminations indicate a hydrostatic environment characterized by low depositional rates and weak sedimentary water dynamics.

Fourthly, By analyzing the trace elements of thin-layered chert and co-occurring thin-layered mud-crystal limestone in the Dalong Formation of the Changjiang Gou section of Guangyuan, Sichuan, we found that V/(V + Ni) ranges from 0.5 to 0.89, V/Cr ranges from 1.03 to 17.50, and Ni/Co ranges from 4.58 to 19.20. These ratios (V/(V + Ni), V/Cr, and Ni/Co) can serve as reliable paleo-anoxic discriminatory markers for environmental conditions (refer to Table 3). The data presented in Tables 3 and 6 demonstrate that the coeval occurrence of thin-layered mud-crystal limestone with the thin-layered chert at the well site in the Dalong Formation of Guangyuan Changjiang Gou section, Sichuan is indicative of anoxic deposition within a reducing environment.

**Table 6:** Criteria for distinguishing geochemical indicators of anoxic and oxygen-rich environments (Hatch and Levenhal, 1992; Jones and Manning, 1994).

Discriminatory indicators	Anoxic environments		Oxygen-rich environment
	Anaerobic	Oxygen-poor	
V(V+Ni)	>0.6	0.46~0.6	<0.46
V/Cr	>4.25	2~4.25	<2
Ni/Co	>7	5~7	<5

In summary, it can be inferred that the depositional environment of the thin-layered chert in the study area likely occurred at a depth exceeding 200 m.

### Genetic mechanisms of nodular, agglomerate, and banded cherts

In contrast to thin-layered cherts, nodular, agglomerate, and banded cherts are products of secondary metasomatism based on their lithology, with their protoliths primarily consisting of limestones from normal shallow marine carbonate terraces. Nodular, agglomerate, and banded cherts occur as isolated lenticular, ellipsoidal, and irregular agglomerates within limestone formations and do not exhibit sedimentary characteristics. These cherts are characterized by the presence of calcareous organisms that undergo accounts and silicification processes [9]. They may form through the co-precipitation of silica material along with carbonate material in seawater during sedimentary diagenesis. The dispersed silica material migrates towards suitable precipitation centers for SiO_2_ growth within the sediment matrix leading to localized enrichment of SiO_2_ content in carbonate rock sediments (rocks), ultimately resulting in the formation of nodular, agglomerate, and banded chert.

The chemical composition analysis of the chert reveals (Table 1) that SiO_2_ dominated the nodular, agglomerate, banded cherts, and thin-layered chert, followed by CaO in bonded form. The agglomerated and banded cherts exhibited an SiO_2_ content of 83.77%, while the CaO content ranged from 0.11% to 26.57%, with an average value of 7.98%. The thin-bedded chert contained 83.77% SiO_2_ and 5.97% CaO, with a relatively low CaO content compared to the combined, agglomerate, and banded cherts; however, it displayed a higher P_2_O_5_ content.

**Fig 9:**
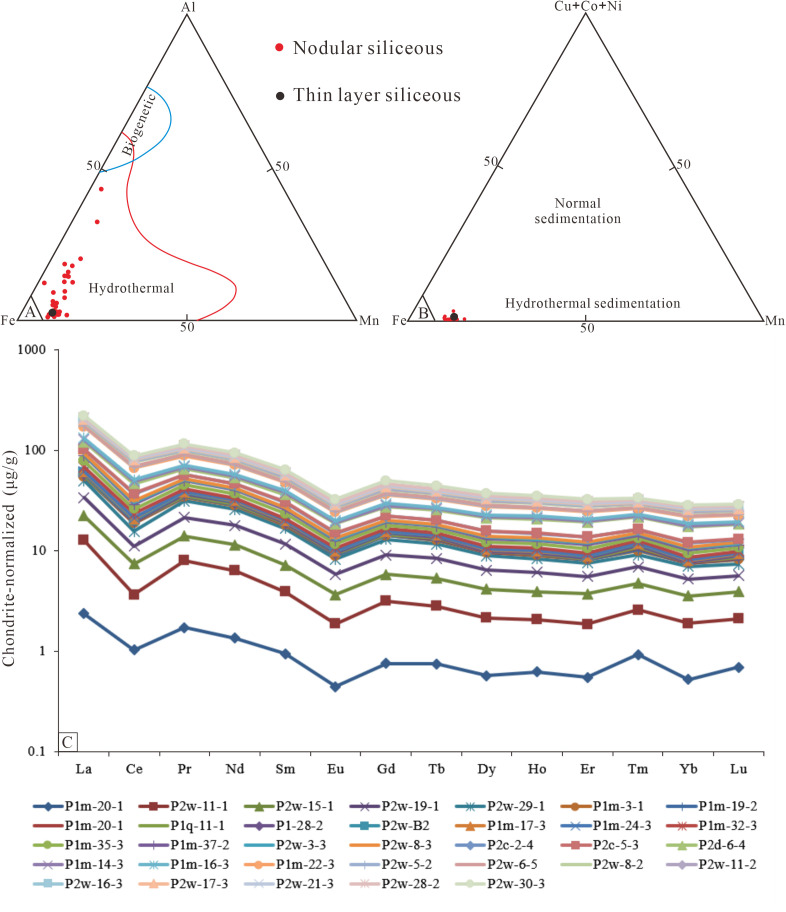
Geochemical characteristics of Permian chert in the Middle-Upper Yangtze region. A- AL-Fe-Mn elemental partitioning diagram. B- (Cu + Co + Ni)-Fe-Mn elemental partitioning diagram. C- REE allotment pattern diagram (spheroidal meteorite standardization).

The Al, Fe, and Mn contents play a crucial role in elucidating the genesis of silica. Typically, the enrichment of Fe and Mn is primarily associated with hydrothermal processes, while that of Al is predominantly linked to clastic materials derived from terrestrial sources. When the Al/(Al + Fe + Mn) ratio falls within the range of 0.01 to 0.43 (with an average value of 0.09), significantly below 0.40, it indicates that the silica in this area originates from hydrothermal activity. This perspective is supported by the Al-Fe-Mn triangles (Fig 9A) and (Cu + Co + Ni)-Fe-Mn triangles (Fig 9B) presented by Chen et al., 1992). Fig 9A and Fig 9B demonstrate that all siliciclastic rocks in this region fall within the hot-water depositional zone.

Deposition and diagenesis exert minimal influence on rare earth elements (REEs) compared to major and trace elements, which better preserve the original information in cherts [3]. The total REE content in Permian chert samples from the Middle-Superior look area ranges from 0.37 to 25.91 μg/g, with a mean value of 6.52 μg/g. Cherts of the Maokou Formation exhibit lower element content and total REE amounts compared to those of the Wujiaping Formation, characterized by LREE/HREE values ranging from 0.16 to 1.06 (mean = 0.65), indicating moderate HREE enrichment and relative LREE deficit. The Ce/Ce* values range from 0.16 to 0.85 (average = 0.53) with a distinct negative anomaly; Eu/Eu* values range from 0.63 to 3.04 (average = 0.94) displaying weak negative or positive anomalies (Table 5).

The standardized REE partitioning pattern of spheroidal meteorites is illustrated in Fig 9-C. Sample P3d-6–4 consists of thin-layered silica, while the remaining samples exhibit nodular, agglomerate, and striped silica (Table 5). Fig 9 demonstrates that all silica rocks within the study area share similar characteristics in terms of their REE distribution patterns, suggesting a common source for silicon elements in both thin-layered and other types of silica rocks. Notably, they all display negative Ce anomalies, indicating their distance from continental environments and absence of terrestrial clastic material influence. This further supports the hypothesis that hydrothermal deposition played a role in supplying silicon elements to these rock formations. Lin et al. (2010) estimated the formation temperature range of siliciclastic rocks in Shizhu Maokou [38], Chongqing based on oxygen isotopes analysis and concluded an average temperature of 67 °C with variations between 34–89 °C for ancient seawater during siliciclastic rock formation. These findings also suggest a significant impact of hot-water deposition on the formation process.

Furthermore, the cherts of the Maokou Formation exhibit a relatively consistent REE content around 0.01 μg/g, indicating low total REE and HREE enrichment. Conversely, the cherts of the Wujiaping Formation display a varying REE content around 0.1 μg/g, suggesting relatively high total REE content but lower HREE enrichment. These observations imply that hydrothermal activity is more pronounced during the Maokou Formation compared to the Wujiaping Formation. Murray et al. (1992) demonstrated that an increased Eu/Eu* value in F-MH chert near mid-ocean ridges signifies stronger hot-water influence. In our study area, the average Eu/Eu* value for cherts was determined as 0.94 [39], highlighting predominant influences from hydrothermal processes and normal seawater during their formation.

### Hydrothermal and chert enrichment

The term ‘hydrothermal’ was initially defined as the hydrothermal and mineralization associated with magmatic activity. White (1974) acknowledged the widespread usage of this term and expanded its definition to encompass “aqueous solutions that are warmer and more concentrated than their surrounding environment.” [40] Conversely, Chen et al. (1992) argued against specifying a uniform constant temperature or temperature range as the lower limit for hydrothermal, instead proposing a significantly higher temperature compared to the local background temperature as the defining criterion [41]. In 1999, The Geological Society of America defined “hydrothermal” as aqueous solutions with a temperature range of 50–700 °C, typically below 400 °C, and a pressure range of 1–3 kPa. Recent global investigations into definitions of ‘hydrothermal’ have revealed its association with magma. Numerous contemporary editions of geochemical terminology define hydrothermal as an aqueous solution formed through natural geological processes at temperatures higher than those found on its ambient surface, originating from either a single source or multiple sources.

Depending on the environment, hydrothermal activity can be classified into marine and terrestrial settings [42–46]. The marine environment is primarily influenced by seawater and magmatic water as the main sources of hydrothermal fluids [47–49]. The composition of these fluids exhibits significant variations due to their original source environments. Seawater’s chemical composition remains stable, but its transformation into a hot-water fluid encompasses a wide range of comparable types, leading to the formation of chemically diverse deposits. Many substances’ solubility is temperature- and pressure-dependent, particularly ions or compounds such as K + , Na + , B_2_O_3_, SiO_2_, Al_2_O_3_, Cl-, F-, etc., along with certain metallic elements [7,16,23]. Consequently, high-temperature hydrothermal fluids possess complex compositions where numerous compounds tend to reach saturation levels. This allows for the sequential development of various sedimentary zonations or layering as temperature and pressure decrease. In our study area, cherts are inferred to have formed through nodular aggregation and banding processes when SiO_2_ becomes saturated in hydrothermal solutions at lower temperatures and pressures or precipitates from seawater. These processes result in localized enrichment of SiO_2_ within carbonate rock sediments.

## Conclusion

Permian laminated and unstratified cherts are well-developed in the Upper Yangtze region, exhibiting distinct lithological and geochemical characteristics that provide insights into their depositional environment and genesis. Conversely, the various unstratified cherts primarily originate from the dissolution of siliceous carbonate rocks by hydrothermal fluids and seawater, thus representing significant examples for understanding the genesis of such non-laminated siliceous deposits.

The relatively homogeneous mineralogical composition of cherts has hindered the advancement of research on cherts compared to clastic and carbonate rocks. Although research on cherts was concentrated in the late 20th century, international interest and studies in this field have significantly declined in recent years. While the primary focus has been on understanding the early Formations of Earth formation, further investigation into the microscopic composition and diagenetic processes of cherts is necessary. Moreover, there is a notable lack of research on isotopes in cherts and their related applications, despite their potential as indicators for genesis and geotectonic settings; thus requiring further exploration.
